# Comparative Analysis of *Campylobacter jejuni* and *C. coli* Isolated from Livestock Animals to *C. jejuni* and *C. coli* Isolated from Surface Water Using DNA Sequencing and MALDI-TOF

**DOI:** 10.3390/pathogens12091069

**Published:** 2023-08-22

**Authors:** Martine Denis, Valérie Rose, Bérengère Nagard, Amandine Thépault, Pierrick Lucas, Meagan Meunier, Fabienne Benoit, Amandine Wilhem, Benoit Gassilloud, Elodie Cauvin, Alain Rincé, Michèle Gourmelon

**Affiliations:** 1 Ploufragan-Plouzané-Niort Laboratory, Hygiene and Quality of Poultry and Pork Products Unit, ANSES (French Agency For Food, Environmental and Occupational Health and Safety), 22440 Ploufragan, France; valerie.rose@anses.fr (V.R.); berengere.nagard@anses.fr (B.N.); amandine.thepault@anses.fr (A.T.); 2 Ploufragan-Plouzané-Niort Laboratory, Viral Genetics and Biosafety Unit, ANSES, 22440 Ploufragan, France; pierrick.lucas@anses.fr; 3 Caen-Saint-Lô Laboratory, Research Department, LABEO, 50000 Saint-Lô, France; meagan.meunier@laboratoire-labeo.fr (M.M.); fabienne.benoit@laboratoire-labeo.fr (F.B.); elodie.cauvin@laboratoire-labeo.fr (E.C.); 4 Nancy Laboratory, ANSES, PTF Maldi, 54000 Nancy, France; amandine.wilhem@anses.fr (A.W.); benoit.gassilloud@anses.fr (B.G.); 5 Bacterial Communication and Anti-Infectious Strategies Reseach Unit, UNICAEN (Caen Normandie University), UR4312 CBSA, 14000 Caen, France; alain.rince@unicaen.fr; 6 ODE-DYNECO-PELAGOS (Department of Oceanography and Ecosystem Dynamics, Coastal Environment Dynamics and Pelagic Ecology Research Unit), IFREMER (French Research Institute for Exploitation of the Sea), 29280 Plouzané, France; michele.gourmelon@ifremer.fr

**Keywords:** *Campylobacter*, livestock animals, surface water, MLST, cgMLST, structure, MALDI-TOF

## Abstract

This study evaluated the contribution of cattle, sheep, poultry and pigs to the contamination of surface water from rivers by *Campylobacter jejuni* and *C. coli* using MLST, cgMLST and considered MALDI-TOF MS as an alternative technique. The 263 strains isolated from cattle (n = 61), sheep (n = 42), poultry (n = 65), pigs (n = 60) and surface water (n = 35) were distributed across 115 sequence types (STs), 49 for *C. jejuni* and 66 for *C. coli.* Considering MLST data, 14.2%, 11.4% and 2.8% of the surface water strains could be attributed to cattle, poultry and sheep, respectively, none to pigs, and 85.7% were non-attributed. Analysis of cg-MLST data with STRUCTURE indicated that *C. jejuni* strains from water were predominantly attributed to poultry (93.5%), weakly to sheep (<1%) and 6.3% non-attributed, and that conversely, *C. coli* strains from water were predominantly non-attributed (94.3%) and 5.7% attributed to poultry. Considering the protein profiles with a threshold of 94% and 97% of similarity, respectively, strains from surface water could be attributed to poultry (31.4% and 17.1%), and to cattle (17.1% and 5.7%); 54.1% and 77.1% were non-attributed. This study confirmed these livestock animals might contribute to the contamination of surface water, with a level of contribution depending on the typing technique and the method of analysis. MALDI-TOF could potentially be an alternative approach for source attribution.

## 1. Introduction

*Campylobacter* spp. is one of the leading causes of foodborne illness in France, with an estimated 330,000 to 1,060,000 cases of campylobacteriosis each year [1], with *Campylobacter jejuni* (83.4%) and *C. coli* (13.6%) being the two species most frequently isolated in human cases [2]. 

This pathogen can be isolated from a wide range of sources including retail meats, livestock, wild birds, environmental reservoirs and pets [3,4,5,6]. Western France is a region of high livestock production for meat (pork, poultry, beef, veal and sheep), dairy (cows) and eggs (hens). Many farms and slaughterhouses are concentrated in this geographical area. These livestock species are known to be reservoirs of *Campylobacter* spp., carrying it asymptomatically in their digestive tracts. 

The prevalence in *Campylobacter* spp. in these livestock production sectors is high in France. For example, reports show that 70 to 100% of the batches of poultry arriving at the slaughterhouse carry *Campylobacter* spp. [4,7]. *Campylobacter* spp. has been detected in the faeces of pigs on 69% [3] to 100% [8] of pig farms. A recent study on faeces at the slaughterhouse yielded an individual prevalence of 39.3% for cattle and 99.4% for calves [9]. A survey realised in 2017 in 14 cattle farms and 12 sheep farms revealed that the occurrence of *Campylobacter* on cattle and sheep farms was high with 86% _95%CI_ [70–101%] and 83% _95%CI_ [65–101%] of the cattle farms and sheep farms having *Campylobacter* spp.-positive samples, respectively [10]. 

*Campylobacter* spp. is excreted at relatively high rates by animals: around 10^8^ CFU/g, 5.10^5^ CFU/g and 3.10^6^ CFU/g of faeces in poultry [4], pigs [8] and sheep [11], respectively. The spreading of manure, slurry and livestock effluents and animals grazing on pastures can contribute to the spread of *Campylobacter* spp. in the environment and indirectly to the contamination of surface water by runoff. 

This pathogen can survive for a very long time in surface water and domestic wastewater [12]. *Campylobacter* spp. can be detected throughout the year in the water of four rivers in Brittany, France [13]. A recent French study found *Campylobacter* in river water flowing into estuaries and seawater, with a prevalence of 88.6% for river water and of 58.3% for seawater [14]. These *Campylobacter* spp. present in surface waters can originate from animals or humans. The precise identification of the source(s) of *Campylobacter* in surface water and the level of their contribution require strain typing. 

Among available typing methods, multilocus sequence typing (MLST) and core genome MLST (cgMLST) are effective ways to determine the origin of human infections and also the origin of surface water contamination, with *Campylobacter* spp. [15,16,17,18,19,20]. However, these techniques are time-consuming and costly and require bioinformatics skills. 

Two studies tested MALDI-TOF as bacterial typing method by considering the protein profiles (MALDI-Types) of bacteria. The first one [21] associated MALDI-types with sequence types (STs) of *Campylobacter* spp. The second one [22] demonstrated that protein profiles allowed to predict *C. jejuni* clonal complexes (CCs) and STs, at 100% and 93%, respectively. This method has the advantage of being simple because it requires a simple deposit of protein extracts of bacteria and few minutes to obtain the protein profiles. 

The aims of this study were to evaluate the contribution of cattle, pig, poultry and sheep in surface water contamination using MLST, cgMLST and MALDI-TOF, and see if MALDI-TOF can be an alternative technique to answer this question. Here, we focused on *Campylobacter jejuni* and *Campylobacter coli*, the two species most frequently isolated from these livestock animals and from surface water [14]. 

## 2. Materials and Methods

### 2.1. Collections of Strains

The *C. jejuni* and *C. coli* strains considered in this study were isolated from different sources: pigs (n = 60), cattle (n = 61), sheep (n = 42), poultry (n = 65) and surface water of rivers (n = 35) (Table 1). 

These strains were selected from previous collections (pigs, poultry and surface water) or from sampling carried out in 2017 for this project (cattle and sheep). The number of *C. jejuni* and *C. coli* per source selected for typing was proportional to the distribution of these two species in the initial collections, assuming that it is the same proportions reaching the water surface of rivers. With the exception of surface water, we typed all the isolated strains from surface water. All the *Campylobacter* strains considered in this study were isolated in France.

Poultry and pig strains come from national surveys carried out in slaughterhouses in 2012 (not published). All pig strains were *C. coli* (100%), 55.4% and 44.6% of poultry strains were *C. jejuni* and *C. coli*, respectively. They were isolated from the caeca of poultry and from the colon contents of pigs taken at random from slaughter lines at different times of the year. Most of them came from conventional poultry farms and pig farms and some on which animals had access to open areas. All the strains in this study were from the slaughterhouses located in western France. 

Cattle and sheep strains were isolated from a survey carried out in 2017 on 14 cattle farms and 12 sheep farms [10]. The area of sampling focused on the Regnéville-sur-Mer and La Vanlée bays on the western Normandy coast. The cattle and sheep farms were mostly chosen for their proximity to these bays, except two sheep farms, which were located further north, near Saint-Germain-sur-Ay bay. The strains were isolated from pools of faeces (10 pools of faeces per farm) sampled on the ground at different farm locations. The strains of *Campylobacter* recovered from cattle faeces were mainly *C. jejuni* (98%), the others being *C. hyointestinalis*. The strains collected from sheep faeces were mostly *C. jejuni* (66%), the others being *C. coli* (34%). 

The strains sampled from surface water were collected from 2013 to 2015 [14]. These rivers flow into the La Fresnaye Bay in the north of Brittany or into Régneville-sur-Mer and La Vanlée bays on the western Normandy coast, France. 

### 2.2. MLST and cgMLST

Genomic DNA was extracted from fresh colonies using the QIAamp DNA mini kit from QIAGEN and genomes were sequenced at ICM (Paris, France) using Illumina MiSeq technology. Genomes were assembled using the Shovill pipeline.

The analysis of the traditional seven-gene MLST was performed using whole-genome sequencing (WGS) data by comparing the sequences with the publicly available PubMLST database (BIGSDB, http://pubmlst.org/campylobacter (accessed on 1 March 2019)). Simpson’s diversity index (ID) was calculated using the online tool “Comparing Partitions” at http://www.comparingpartitions.info (accessed on 1 March 2019). 

The cgMLST types were determined according to the 1343-locus cgMLST scheme [23]. We considered only the loci common to all genomes in each species. Finally, up to 1100 and 1108 loci were run for cgMLST for *C. jejuni* and *C. coli*, respectively. 

All the sequence analyses were performed using BioNumerics software (version 7.5) (Applied Maths NV; Sint-Martens-Latem, Belgium), with graphical representations of the population plotted as minimum spanning trees (MST) based on the MLST and cgMLST data. A threshold of 11 allelic differences was used to classify the strains using the Oxford cgMLST scheme, as previously described [24].

### 2.3. Source Attribution Using STRUCTURE

Source attribution of strains of *C. jejuni* (n = 14) and *C. coli* (n = 20) recovered from surface water was performed using STRUCTURE software, publicly accessible online at this address https://web.stanford.edu/group/pritchardlab/structure.html (accessed on 1 March 2023). STRUCTURE includes a clustering method based on a Bayesian model enabling population structure inference and attribution of individuals to sources using genotyping data [25]. Probabilistic assignments were carried out separately for *C. jejuni* and *C. coli*, using allelic profiles of 1341 core-genome loci for *C. jejuni* and 1328 core-genome loci for *C. coli.* The “no admixture” model was used with a correlated allele frequency model and assumed that each isolate originated in one of the putative source populations showing each a characteristic set of allelic frequencies [26]. Analyses were performed with 10,000 burn-in cycles followed by 10,000 iterations, consistent with previously published work [27]. The reference data set used for *C. jejuni* source attribution included 61 bovine strains, 36 chicken strains, and 27 sheep strains. Regarding *C. coli* source attribution, the reference data set comprised 59 swine strains, 29 chicken strains and 15 sheep strains. For both analyses a fourth putative source was created to represent unsampled hosts/reservoirs such as wild animals, companion animals, etc. in accordance with the approach previously published [28].

### 2.4. MALDI-Typing

Strains were subcultured on blood agar for 24 h at 37 °C in microaerobic conditions. The proteins of each strain were extracted as recommended by Bruker. Colonies were suspended in 300 µL of water for injection (WFI) in which 900 µL of pure ethanol were added. After a centrifugation at 13,000 rpm for 2 min, the pellets were then mixed with 10 µL of formic acid 70% and then with 10 µL of acetonitrile. After another centrifugation at 13,000 rpm for 2 min, 1 µL of the supernatant was deposited eight times per strain on a plate of MSP 48 target polished steel. After drying, 1 µL of IVD-HCCA (α-Cyano-4-hydroxycinnamic acid) matrix was added on each target. The protein extracts were sent to MALDI-TOF Platform of the Anses. Mass spectra were acquired on a Microflex LT mass spectrometer (MALDI Biotyper from Bruker Daltonics). The default parameters were: detection in linear positive mode, laser frequency of 60 Hz, ion source voltages of 2.0 and 1.8 kV, lens voltage of 6 kV) within the *m*/*z* of 2000–20,000. For each strain, 32 spectra per protein extract (4 reads per target with 8 targets per strain) was obtained. External calibration of the mass spectra was performed using Bruker Bacterial Test Standard (BTS) (8255343, Bruker Daltonics GmbH, Bremen, Germany).

The spectra were preprocessed by applying the “smoothing” and “baseline subtraction” procedures available in FlexAnalysis software (version 3.4.79.0) (Bruker Daltonics GmgH, Bremen, Germany). 

The spectra were exported as peak lists with *m*/*z* values and signal intensities for each peak in text format and then imported into a database in BioNumerics software (version 7.5) (Applied Maths NV; Belgium). Peak detection was performed in BioNumerics using a signal-to-noise ratio of 20. An average spectrum of protein for each strain was created using the following parameters: minimum similarity, 90%; minimum peak detection rate, 60%; constant tolerance, 1; and linear tolerance, 300 ppm. Then all the average spectra were compared in a circular tree using the UPGMA method and Pearson’s coefficient. 

A previous study using a similarity threshold of 94% showed that protein profiles can predict CC and ST using a random forest algorithm [21]. Although our approach to analysing the results is different, we have taken this threshold as a reference and tested a lower and a higher threshold. We considered the results according to three similarity thresholds ≥to 91%, ≥to 94%, ≥to 97%.

## 3. Results

### 3.1. Contribution of Livestock Type to Surface Water Contamination with Genome Comparison

The 263 strains were distributed across 115 STs: 49 for *C. jejuni* and 66 for *C. coli*, the most prevalent being ST19 and ST21 for *C. jejuni*, and ST854 and ST2699 for *C. coli*.

The diversity of all our *C. jejuni* collection was high (ID = 0.94, _95%CI_ [0.93–0.97]); while this diversity was lower in cattle and sheep than in poultry (Table 2). Among the 138 *C. jejuni* strains, 49 STs were identified: 16 in cattle, seven in sheep, 25 in poultry and 11 in surface water (Table 2). These STs were distributed into 20 clonal complexes (CC); 18 strains (11 STs) had unassigned CCs. Two CCs were predominant, with CC21 and CC45 representing 30.4% (n = 42) and 10.1% (n = 14) of the strains, respectively. Several STs of *C. jejuni* were assigned to a single source: 10 of the 16 STs found in cattle, five of the seven STs in sheep, 20 of the 25 STs in poultry and eight of the 11 STs in surface water. 

Only six STs included *C. jejuni* strains from different sources: ST19 and ST21 (=CC21), ST45 and ST137 (=CC45), ST38 and ST257 (Table 2). Strains from cattle (57.3%) had STs in common with strains from sheep (44.4%) and/or poultry (19.4%). One strain isolated from water clustered in ST19 with 14 cattle strains, seven sheep strains and one poultry strain. Three other strains from water clustered in ST45 with three cattle strains and one poultry strain, and one water-sourced strain clustered in ST137 with one cattle strain. Finally, 35.7%, 28.5% and 7.14% of *C. jejuni* strains from water had similar STs to strains isolated from cattle, poultry and sheep, respectively (Table 3(a)), and nine (64.3%) were not attributed to any animal source.

The diversity of all *C. coli* collection was also high (ID = 0.96; _95%CI_ [0.93–0.98]) with a lower diversity in sheep than in the other livestock species (Table 2). Among the 125 *C. coli* strains, 66 STs were identified (33 in pigs, four in sheep, 21 in poultry and 17 in surface water), with 43 STs in CC828 (the only CC identified for *C. coli* in this study). Only two of the 21 strains isolated from surface water carried CC828; the 23 other STs had unassigned CCs (Table 2). Three STs were predominant: ST854 (22 strains), ST2699 (11 strains) and ST825 (seven strains). Several STs were assigned to a single source; 27 of the 33 STs were identified in pigs, one of the four STs in sheep, 15 of the 21 STs in poultry and all 17 STs in surface water.

Only six STs of *C. coli* grouped strains from different sources (ST825, ST854, ST890, ST1016, ST1096 and ST2699), indicating that strains from pigs (40%) had STs in common with strains from sheep (93.3%) and/or poultry (52.4%) (Table 2). However, none of the 21 *C. coli* strains isolated from surface water shared an ST in common with other sources indicating that 100% were not attributed to any animal source (Table 3(b)).

**Figure 1:**
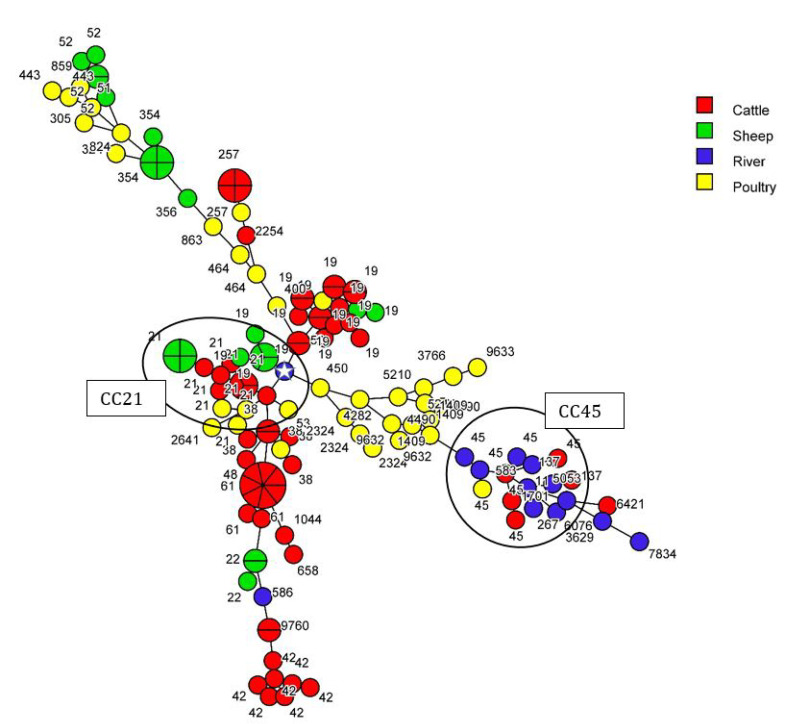
cgMLST-based MST (1100 loci) of *C. jejuni* isolated from cattle, sheep, poultry and river samples. Complex clonal CC21 and CC45 are represented on the MST and ST of the strains are indicated. Taking into account an allelic difference of 11 for the clustering of the strains in cgMLST, only one *C. jejuni* strain from water (identified by a white star) could be linked to strains from sheep (7 allele differences). The number of sections in a circle corresponds to the number of strains.

**Figure 2:**
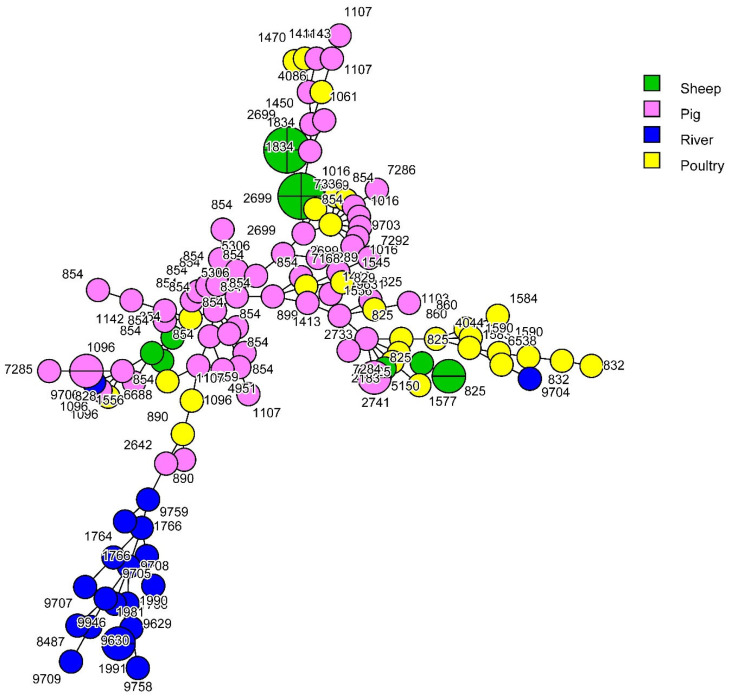
cgMLST-based MST (1108 loci) of *C. coli* isolated from pig, sheep, poultry and river samples. Taking into account an allelic difference of 11 for the clustering of the strains in cgMLST, no *C. coli* strain from any animal source clustered with any *C. coli* water strain.

Taking into account an allelic difference of 11 for the clustering of the strains in cgMLST, no animal strain from any source clustered with any other animal strain. Only one *C. jejuni* strain from water (2.8%) could be attributed to sheep, and none to cattle, poultry or pigs (Figure 1 and Figure 2) indicating that 92.8 % of the *C. jejuni* from water and that 100% of the *C. coli* from water were not attributed to any animal source by cgMLST. 

### 3.2. Source Attribution of C. jejuni and C. coli Strains Retrieved from Surface Water

Probabilistic assignments of the surface water strains to each potential host source were performed separately for *C. jejuni* and *C. coli* using STRUCTURE software, publicly accessible online at this address https://web.stanford.edu/group/pritchardlab/structure.html (accessed on 1 March 2023) and allelic profiles of core-genome loci. A total of 124 agricultural *C. jejuni* strains and 103 agricultural *C. coli* strains were used as reference data sets to attribute surface water *C. jejuni* (n = 14) and *C. coli* (n = 21) strains to their source. 

A total of 93.5% of *C. jejuni* surface water isolates were attributed to poultry, while 6.3% were attributed to unsampled sources and the remaining (<1%) to the sheep reservoir (Table 4). Cattle did not appear to contribute to surface water contamination by *C. jejuni* in this study. Regarding *C. coli*, 94.3% of surface water isolates were attributed to unsampled sources, while 5.7% were attributed to poultry (Table 4). Two sources did not appear to contribute to the surface water contamination by *C. coli*—pig and sheep reservoirs.

### 3.3. Contribution of Animal Production in the Surface Water Contamination with MALDI Typing Comparison

**Figure 3:**
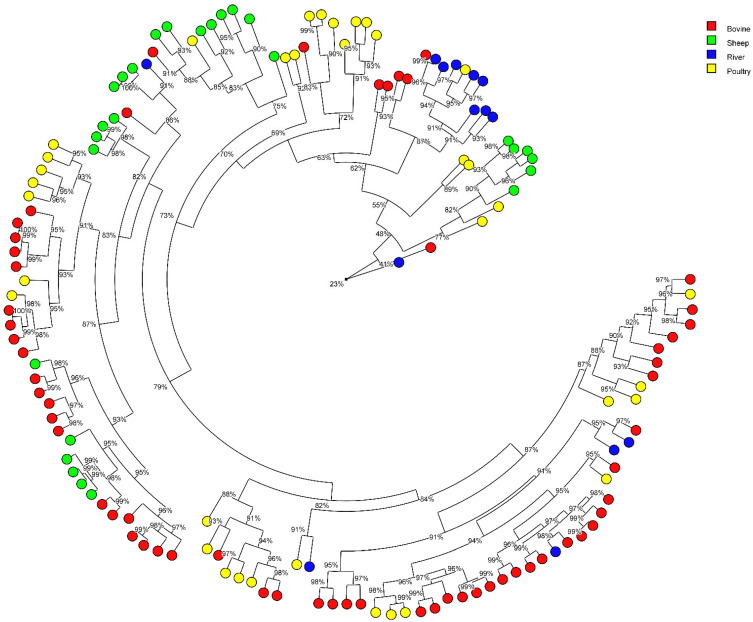
Radial tree of *Campylobacter jejuni* isolates from their protein average spectrum obtained by MALDI-TOF. The radial tree was created with BioNumerics^®^ software (version 7.5) (Applied Maths NV; Belgium) using UPGMA method and Pearson’s coefficient. Clusters were considered from a cut-off ≥to 91%, 94% and 97% of similarity.

**Figure 4:**
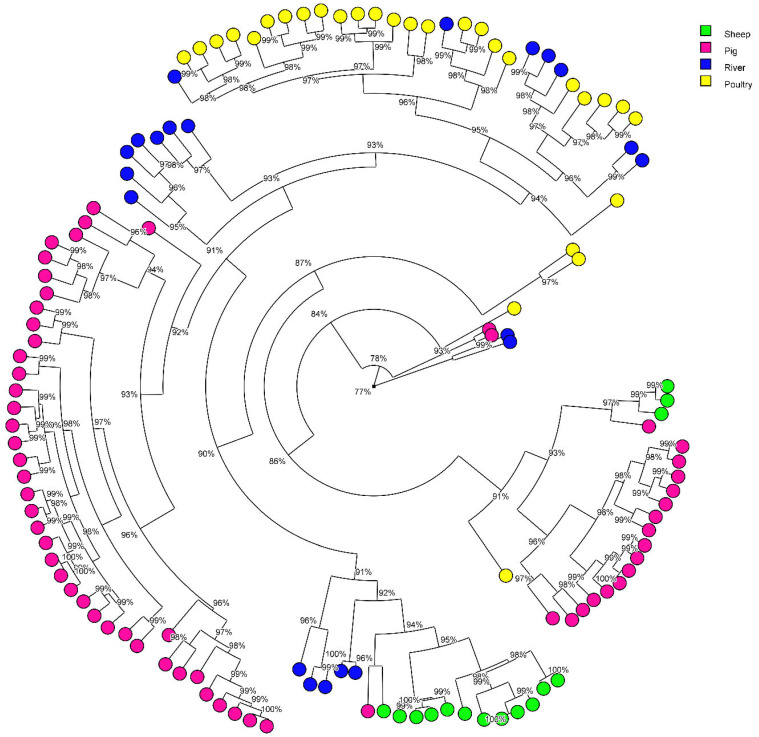
Radial tree of *Campylobacter coli* isolates from their protein average spectrum obtained by MALDI-TOF. The radial tree was created with BioNumerics^®^ software (version 7.5) (Applied Maths NV; Belgium). using UPGMA method and Pearson’s coefficient. Clusters were considered from a cut-off ≥to 91%, 94% and 97% of similarity.

The similarity of the *C. jejuni* and *C. coli* strains according their MALDI-types are represented in two distinct circular trees (Figure 3 and Figure 4, respectively). We analysed the results according to three similarity thresholds ≥to 91%, ≥to 94%, ≥to 97 % (Table 3(a,b)). 

We observed that the *C. jejuni* strains from bovine, poultry and/or sheep can be grouped on the same branches suggesting a close similarity and that few branches were linked to only one host. The percentage of water strains non-attributed to an animal origin increases in parallel with the increase of the threshold, to the detriment of the percentage attributed to cattle and poultry (Table 3(a)). At ≥91%, sheep are identified as a potential source of river contamination by *C. jejuni*, but no longer at 94% and 97%. At ≥91%, only four *C. jejuni* water strains clustered in MALDI with animal strains had the same ST as animal strains (ST19 (1 strain), ST45 (2 strains) and ST137 (1 strain)); the others water strains had no ST in common (ST11, ST267, ST583, ST586, ST3629, ST5053, ST6076, and ST7834) with animal strains. The concordance of results between these two techniques, MLST and MALDI, is therefore weak. 

On the *C. coli* circular tree, strains from pigs, poultry and sheep were almost separated from each other according to the origin, suggesting that these *C. coli* strains are strongly associated with the host. The percentage of water strains non-attributed to an animal origin increases in parallel with the increase of the threshold, to the detriment of the percentage attributed to poultry (Table 3(b)). At ≥91%, pig and sheep are identified as a potential source of river contamination by *C. coli*, but no longer at 94% and 97%. At ≥91%, no *C. coli* water strains clustered in MALDI with animal strains had the same ST as animal strains (ST1764, ST1766, ST1981, ST1990, ST1991, ST8487, ST9629, ST9704, ST9705, ST9706, ST9707, ST9708, ST9709, ST9758, and ST9759). There was no concordance in the results between the two techniques, MLST and MALDI. 

## 4. Discussion

This study focused on a collection of *C. jejuni* and *C. coli* strains isolated in France from different livestock types—cattle, sheep, pigs and poultry—to evaluate the contribution of these farm animals in the contamination of surface water of rivers. First, the overall diversity was high, as was the diversity of strains within each source (with *C. coli* in pigs showing the lowest diversity). The 263 strains were distributed across 115 STs (49 for *C. jejuni* and 66 for *C. coli*), suggesting a good representativeness of the STs occurring in the different sources considered here and providing sufficiently robust data to demonstrate the link between STs and their hosts. For example, several STs were specific to one source whereas other STs were found in several sources. It is common for different animal species to share the same STs [19,29,30]; some STs belonging for example to the ST-21 or ST-45 clonal complexes can be considered as host-generalist STs because they can be isolated from multiple reservoirs [31]. 

### 4.1. Diversity of Sequence Types in Cattle, Sheep, Poultry and Pigs

In cattle, we had mainly *C. jejuni*, whereas *C. coli* was also isolated [9], albeit at a lower level (7.8% of the strains) than *C. jejuni*. *C. coli* has also been isolated from cattle in Spain [32]. As also observed in other studies [9,33,34], CC21 was prevalent in our cattle *Campylobacter* population. Five STs identified only in cattle (ST38, ST42, ST61, ST45 and ST658) were common to STs isolated from bulk tank milk in dairy herds in Italy [34], suggesting that some strains can be host-specific.

In our study, sheep excreted both *C. jejuni* and *C. coli*. These two species are associated with abortion in sheep in the USA [35,36] and in Turkey [37]. The USA study [35] identified ST19 and ST21 as responsible for sheep abortion; these two STs were isolated in our study from healthy sheep. Our strains from sheep were distributed across only 11 STs, with ST2699 being the most prevalent, followed by ST19 and ST21. These two ST belong to CC21, a CC that was prevalent in sheep in a Spanish study [33]. Among the 11 STs identified in sheep in our study, five were also found in cattle, poultry and/or pigs, further supporting the fact that some STs are host-generalist STs.

The diversity of STs from *C. jejuni* and *C. coli* from poultry was high, as already observed for the poultry production sector in France [38,39]. Although those studies highlight the predominance of CC21, CC45 and CC464 in the broiler production chain, there was no particular ST or CC that stood out from the others in our poultry sample collection. 

The only CC found in our study for *C. coli* was CC828 (the others being unidentified), which has been previously described as the predominant CC in *C. coli* isolates [40]. In pigs, we identified *C. coli* only as recently reported in the French pig production sector [3,41]. The diversity of our *C. coli* from pigs was high (33 STs for 60 strains) and ST854 was one of the most prevalent STs as reported in other countries, e.g., Switzerland, the UK, USA, Denmark and Italy [40,42,43,44,45,46]. Nine other STs of our *C. coli* from pigs were also identified in another study [45], indicating that similar strains can be isolated at different times and in different locations. Overall, 40% of the *C. coli* from pigs shared six STs with those from poultry (i.e., 38% of the *C. coli* identified in poultry). The concentration and the proximity of these two livestock types can contribute to the circulation of these STs between them.

### 4.2. Sources of Campylobacter Contamination of Rivers

In our study, CC45 (in particular ST45) was the most prevalent CC found among the strains isolated from the surface water of rivers. This CC is known to be more widely distributed within the environment than other STs [17,47,48]. 

Considering MLST data in our study, the highest contributor to surface water contamination was cattle, followed by poultry and sheep. Pigs were not identified as a source of surface water contamination. This result differs from what has been observed in other countries. Using an asymmetric island model for source attribution on MLST data [17], 4.3% and 1.2% of *C. coli* surface water strains could be attributed to pigs for Luxembourg and the Netherlands, respectively. In these countries, *C. jejuni* and *C. coli* strains from surface water were mainly attributed to wild birds (61.0%, and 37.3%, for respectively, Luxembourg and The Netherlands) and poultry, followed by ruminants and pigs [17]. If we consider all the surface water strains in our study (n = 35), we can estimate that 85.7% are not attributed to any animal production. A proportion of these strains could therefore be attributed to wild birds. However, in a recent study carried out in Luxembourg [49] it was observed that 4% of *C. jejuni* from wild birds corresponded to recurrent genotypes in humans, suggesting a key role for wild birds in the spread and persistence of these genotypes and its multi-host characteristic.

A previous study using a 94% similarity threshold showed that protein profiles can predict CCs and STs with the help of a random forest algorithm [22]. That is why we considered it interesting to type our strains with the MALDI biotyper to see if we reached the same conclusions for these two approaches, MLST and MALDI. Using a threshold of ≥91% of similarity for the protein profiles of the strains, our study showed that strains from surface water were attributed to all the four animal productions and to a greater extent, mainly to poultry, followed by cattle. By raising the threshold to ≥94% and then to ≥97%, we lose the contribution of sheep and pigs. At a threshold of 94%, strains from surface water could be attributed to poultry (31.4%), and to cattle (17.1%), and 54.1% were non-attributed. At a threshold of 97%, strains from surface water could be attributed to poultry (17.1%), and to cattle (5.7%), and 77.1% were non-attributed. This approach may be an alternative method for attributing the origin of strains isolated from surface water or human infections. A threshold of 94% seems to be a good compromise. However, a very low concordance for *C. jejuni* and no concordance for *C. coli* were observed between the MALDI clusters that grouped water strains with animal strains and STs of these water strains; their STs were not found in animals (with the exception of four *C. jejuni* water strains). It is not the same strains that are involved in the attribution between MALDI and MLST. This poor or lack of agreement between the two techniques could be explained by the fact that the protein spectra of strains may be influenced by the environmental conditions they encountered during their lifetime (digestive tract, soil, water, etc.). Therefore, the relevance of MALDI for this type of study is questionable. 

Using cgMLST analysis, the involvement of wild birds mainly and poultry in surface water contamination was confirmed in the Netherlands [20]. Based on 11 allelic differences to cluster the strains in cgMLST [24], we only identified one strain from surface water (2.8%) related to sheep. None of the strains from cattle, poultry or pigs could be associated with strains from river surface water. As a result, 97.2% of the strains isolated from the surface water are not attributed. This could be explained by the fact that cgMLST is more discriminating than MLST, which only considers seven housekeeping genes. Nevertheless, this result suggests that birds make a significant contribution to surface water contamination. 

Of the 35 strains (14 *C. jejuni* and 21 *C. coli*) isolated from surface water, 30 had an ST that was not identified in any of the livestock types. Five STs (ST11, ST267, ST583, ST5053 and ST7834) of our strains isolated from surface water were associated with wild birds in the pubMLST database (consulted 4 July 2023). 

Analysis of cg-MLST data with STRUCTURE indicates that *C. jejuni* strains from surface water are predominantly attributed to poultry (93.5%) with 6.3% non-attributed, and that conversely, *C. coli* strains from surface water are predominantly non-attributed (94.3%) with 5.7% attributed to poultry. Our results are consistent with those obtained previously with STRUCTURE [20] for *C. coli* where 93.7% of the strains in the surface water were attributed to wild birds and 5.6% to poultry. The addition in our analysis of a fourth source to represent unsampled reservoirs such as wild animals, companion animals, etc. as previously described [28] is a very appropriate approach for a better attribution of contamination sources. 

## 5. Conclusions

This study confirmed that these livestock animals—cattle, sheep, poultry and pigs—might contribute to the contamination of surface water. However, our study showed that the level of contribution of each animal production is variable depending on the typing technique and the method of analysis, and that the proportion of non-attributed strains must be taken into account to reflect unsampled sources. Our study suggests that other sources, such as wild birds, would contribute to surface water contamination by *Campylobacter*. MALDI could potentially be an alternative approach for source attribution. However, questions remain about the stability of these protein spectra in different environments and over time. 

## Figures and Tables

**Table 1:** Number of *Campylobacter* strains typed per species and source.

Sources	Pig	Cattle	Sheep	Poultry	River	Total
*C. jejuni*	0	61	27	36	14	138
*C. coli*	60	0	15	29	21	125
Total	60	61	42	65	35	263

**Table 2:** Distribution of *C. jejuni* (on the left) and *C. coli* (on the right) strains according to the sources, Sequence type (ST) and Clonal Complexes (CC).

*Campylobacter jejuni*								*Campylobacter coli*						
CC	ST	Cattle	Sheep	Poultry	River	Total		CC	ST	Pig	Sheep	Poultry	River	Total
21	19	14	7	1	1	23		828	825	2	3	2		7
	21	8	5	3		16			828	2				2
	50	2				2			829			1		1
	53			1		1			832			2		2
22	22		3			3			854	15	3	4		22
42	42	7				7			860			2		2
	9760	2				2			890	1		1		2
45	11				2	2			899	1				1
	45	3		1	3	7			1016	2		1		3
	137	1			1	2			1061	1				1
	583				1	1			1096	2		2		4
	1701	1				1			1103	1				1
	6076				1	1			1107	4				4
48	38	5		1		6			1142	1				1
	48	1				1			1413	2				2
52	52		5			5			1436			1		1
61	61	9				9			1545	1				1
179	6421	1				1			1556	2				2
257	257	4		1		5			1583			1		1
	824			1		1			1590			2		2
	2254	1				1			1834	2				2
283	267				1	1			2183			1		1
353	356		1			1			2642	1				1
	400			1		1			2699	2	8	1		11
	2641			1		1			2733	1				1
354	324			1		1			2741	2				2
	354		5			5			4044			1		1
443	51			1		1			4086			1		1
	443			2		2			5306	2				2
	859			1		1			5959	1				1
446	450			1		1			6538			1		1
464	464			2		2			6688			1		1
574	305			1		1			7168	1				1
607	863			1		1			7285	1				1
658	658	1				1			7286	1				1
	1044	1				1			7288	1				1
952	7834				1	1			7289	1				1
1275	3629				1	1			7292	1				1
NA	586				1	1			7336	1				1
	1409			3		3			9631			1		1
	2274		1			1			9703	1				1
	2324			3		3			9704				1	1
	3766			1		1			9706				1	1
	4282			1		1		NA	1143	1				1
	4490			2		2			1450	1				1
	5053				1	1			1470			1		1
	5210			2		2			1577			1		1
	9632			2		2			1584			1		1
	9633			1		1			1764				2	2
Total		61	27	36	14	138			1766				3	3
ID		0.89	0.84	0.97	0.95	0.94			1981				1	1
95% CI		0.86–0.93	0.80–0.89	0.96–0.99	0.89–1.02	0.93–0.97			1990				1	1
									1991				2	2
									1991				2	2
									4951	1				1
									5150		1			1
									7284	1				1
									8487				1	1
									9629				1	1
									9630				1	1
									9705				1	1
									9707				1	1
									9708				1	1
									9709				1	1
									9758				1	1
									9759				1	1
									9946				1	1
								Total		60	15	29	21	125
								ID		0.93	0.67	0.97	0.98	0.96
								95% CI		0.88–0.98	0.49–0.86	0.95–1.00	0.95–1.01	0.93–0.98

**Table 3:** (a) Attribution of *C. jejuni* strains isolated from water to cattle, sheep and poultry using MLST and MALDI typing with three similarity thresholds ≥to 91%, ≥to 94%, ≥to 97%. Water strains with the same ST or grouped in the same MALDI cluster as animal strains were first considered. The remaining water strains were defined as non-attributed. (b) Attribution of *C. coli* strains isolated from water to pig, sheep and poultry using MLST and MALDI typing with three similarity thresholds ≥to 91%, ≥to 94%, ≥to 97%. Water strains with the same ST or grouped in the same MALDI cluster as animal strains were first considered. The remaining water strains were defined as non-attributed.

(a)		**MLST**			**MALDI ≥ 91%**			**MALDI ≥ 94%**			**MALDI ≥ 97%**	
sources of *C. jejuni* strains	n. of isolates	n. of water strains with same ST as animal strains	% of attributed water strains		n. of water strains in same cluster ≥ 91% as animal strains	% of attributed water strains		n. of water strains in same cluster ≥ 94% as animal strains	% of attributed water strains		n. of water strains in same cluster ≥ 97% as animal strains	% of attributed water strains
Cattle	61	5	35.7		11	78.6		6	42.8		2	14.3
Sheep	27	1	7.1		1	7.1		0	0		0	0
Poultry	36	4	28.5		12	85.7		4	14.3		1	7.1
Water	14	9 non-attributed	64.3		1 non-attributed	7.1		6 non-attributed	42.8		11 non-attributed	78.8
(b)		**MLST**			**MALDI ≥ 91%**			**MALDI ≥ 94%**			**MALDI ≥ 97%**	
sources of *C. coli* strains	n. of isolates	n. of water strains with same ST as animal strains	% of attributed water strains		n. of water strains in same cluster ≥ 91% as animal strains	% of attributed water strains		n. of water strains in same cluster ≥ 94% as animal strains	% of attributed water strains		n. of water strains in same cluster ≥ 97% as animal strains	% of attributed water strains
Pig	60	0	0		5	23.8		0	0		0	0
Sheep	15	0	0		5	23.8		0	0		0	0
Poultry	29	0	0		14	66.6		7	33.3		5	23.8
Water	21	21 non-attributed	100		2 non-attributed	9.52		14 non-attributed	66.6		16 non-attributed	76.2

**Table 4:** Probabilistic assignments of *Campylobacter jejuni* or *Campylobacter coli* isolates recovered from surface water to their source using STRUCTURE software. The values indicated in the table correspond to the probability that *C. jejuni* or *C. coli* isolates recovered from surface water in France, originally come from cattle, poultry, sheep, pig or from an unsampled source.

Sources	Cattle	Pig	Poultry	Sheep	Unsampled
*C. jejuni* (n = 14)	0	NA	0.935	0.003	0.063
*C. coli* (n = 21)	NA	0	0.057	0	0.943

## Data Availability

All data are provided in the results section of this paper and in figures and tables. The reads are accessible in ENA in BioProject PRJEB59116.

## References

[1] Van Cauteren D., De Valk H., Sommen C., King L.A., Jourdan-Da Silva N., Weill F.X., Le Hello S., Mégraud F., Vaillant V., Desenclos J.C. (2015). Community Incidence of Campylobacteriosis and Nontyphoidal Salmonellosis, France, 2008–2013. Foodborne Pathog. Dis..

[2] CNCH (Centre National de Référence des Campylobacters et des Helicobacters), SPF (Santé Publique France) (2020). Rapport de l’Année d’Exercice 2019.

[3] Denis M., Henrique E., Chidaine B., Tircot A., Bougeard S., Fravalo P. (2011). *Campylobacter* from sows in farrow-to-finish pig farms: Risk indicators and genetic diversity. Vet. Microbiol..

[4] Allain V., Chemaly M., Laisney M.J., Rouxel S., Quesne S., Le Bouquin S. (2014). Prevalence of and risk factors for *Campylobacter* colonisation in broiler flocks at the end of the rearing period in France. Br. Poult. Sci..

[5] Thépault A., Méric G., Rivoal K., Pascoe B., Mageiros L., Touzain F., Rose V., Béven V., Chemaly M., Sheppard S.K. (2017). Genome-wide identification of host-segregating epidemiological markers for source attribution in *Campylobacter jejuni*. Appl. Environ. Microbiol..

[6] Thépault A., Rose V., Queguiner M., Chemaly M., Rivoal K. (2020). Dogs and Cats: Reservoirs for Highly Diverse *Campylobacter jejuni* and a Potential Source of Human Exposure. Animals.

[7] Hue O., Le Bouquin S., Laisney M.J., Allain V., Lalande F., Petetin I., Rouxel S., Quesne S., Gloaguen P.Y., Picherot M. (2010). Prevalence of and risk factors for *Campylobacter* spp. contamination of broiler chicken carcasses at the slaughterhouse. Food Microbiol..

[8] Fosse J., Seegers H., Magras C. (2009). Prevalence and risk factors for bacterial food-borne zoonotic hazards in slaughter pigs: A review. Zoonosis Public Health.

[9] Thépault A., Poezevara T., Quesne S., Rose V., Chemaly M., Rivoal K. (2018). Prevalence of Thermophilic *Campylobacter* in Cattle Production at Slaughterhouse Level in France and Link Between *C. jejuni* Bovine Strains and Campylobacteriosis. Front. Microbiol..

[10] Cauvin E., Benoit F., Denis M. Prevalence of *Campylobacter* spp. in cattle and sheep farms, in Normandy, France. Proceedings of the IAFP Congress.

[11] Yang R., Jacobson C., Gardner G., Carmichael I., Campbell A.J., Ryan U. (2014). Longitudinal prevalence, faecal shedding and molecular characterisation of *Campylobacter* spp. and *Salmonella enterica* in sheep. Vet. J..

[12] Jones K. (2001). Campylobacters in water, sewage and the environment. Symp. Ser. (Soc. Appl. Microbiol.).

[13] Denis M., Tanguy M., Chidaine B., Laisney M.J., Mégraud F., Fravalo P. (2011). Description and sources of contamination by *Campylobacter* spp. of river water destined for human consumption in Brittany, France. Pathol. Biol..

[14] Rincé A., Balière C., Hervio-Heath D., Cozien J., Lozach S., Parnaudeau S., Le Guyader F.S., Le Hello S., Giard J.C., Sauvageot N. (2018). Occurrence of Bacterial Pathogens and Human Noroviruses in Shellfish-Harvesting Areas and Their Catchments in France. Front. Microbiol..

[15] Clark C.G., Taboada E., Grant C.C., Blakeston C., Pollari F., Marshall B., Rahn K., Mackinnon J., Daignault D., Pillai D. (2012). Comparison of molecular typing methods useful for detecting clusters of *Campylobacter jejuni* and *C. coli* isolates through routine surveillance. J. Clin. Microbiol..

[16] Jonas R., Kittl S., Overesch G., Kuhnert P. (2015). Genotypes and antibiotic resistance of bovine *Campylobacter* and their contribution to human campylobacteriosis. Epidemiol. Infect..

[17] Mughini-Gras L., Penny C., Ragimbeau C., Schets F.M., Blaak H., Duim B., Wagenaar J.A., de Boer A., Cauchie H.M., Mossong J. (2016). Quantifying potential sources of surface water contamination with *Campylobacter jejuni* and *Campylobacter coli*. Water Res..

[18] Mossong J., Mughini-Gras L., Penny C., Devaux A., Olinger C., Losch S., Cauchie H.M., van Pelt W., Ragimbeau C. (2016). Human Campylobacteriosis in Luxembourg, 2010–2013: A Case-Control Study Combined with Multilocus Sequence Typing for Source Attribution and Risk Factor Analysis. Sci. Rep..

[19] Hsu C.H., Harrison L., Mukherjee S., Strain E., McDermott P., Zhang Q., Zhao S. (2020). Core Genome Multilocus Sequence Typing for Food Animal Source Attribution of Human *Campylobacter jejuni* Infections. Pathogens.

[20] Mulder A.C., Franz E., de Rijk S., Versluis M.A.J., Coipan C., Buij R., Müskens G., Koene M., Pijnacker R., Duim B. (2020). Tracing the animal sources of surface water contamination with *Campylobacter jejuni* and *Campylobacter coli*. Water Res..

[21] Zautner A.E., Masanta W.O., Tareen A.M., Weig M., Lugert R., Groß U., Bader O. (2013). Discrimination of multilocus sequence typing-based *Campylobacter jejuni* subgroups by MALDI-TOF mass spectrometry. BMC Microbiol..

[22] Feucherolles M., Nennig M., Becker S.L., Martiny D., Losch S., Penny C., Cauchie H.M., Ragimbeau C. (2021). Investigation of MALDI-TOF Mass Spectrometry for Assessing the Molecular Diversity of *Campylobacter jejuni* and Comparison with MLST and cgMLST: A Luxembourg One-Health Study. Diagnostics.

[23] Cody A.J., Bray J.E., Jolley K.A., McCarthy N.D., Maiden M.C.J. (2017). Core Genome Multilocus Sequence Typing scheme for stable, comparative analyses of *Campylobacter jejuni* and *C. coli* human disease isolates. J. Clin. Microbiol..

[24] Nennig M., Llarena A.K., Herold M., Mossong J., Penny C., Losch S., Tresse O., Ragimbeau C. (2021). Investigating Major Recurring *Campylobacter jejuni* Lineages in Luxembourg Using Four Core or Whole Genome Sequencing Typing Schemes. Front. Cell. Infect. Microbiol..

[25] Pritchard J.K., Stephens M., Donnelly P. (2000). Inference of population structure using multilocus genotype data. Genetics.

[26] Pritchard J.K., Wen X., Falush D. (2010). Documentation for Structure Software, Version 2.3.

[27] Lévesque S., Fournier E., Carrier N., Frost E., Arbeit R.D., Michaud S. (2013). Campylobacteriosis in urban versus rural areas: A case-case study integrated with molecular typing to validate risk factors and to attribute sources of infection. PLoS ONE.

[28] McLure A., Smith J.J., Firestone S.M., Kirk M.D., French N., Fearnley E., Wallace R., Valcanis M., Bulach D., Moffatt C.R.M. (2023). Source attribution of campylobacteriosis in Australia, 2017–2019. Risk Anal..

[29] Ogden I.D., Dallas J.F., MacRae M., Rotariu O., Reay K.W., Leitch M., Thomson A.P., Sheppard S.K., Maiden M., Forbes K.J. (2009). *Campylobacter* excreted into the environment by animal sources: Prevalence, concentration shed, and host association. Foodborne Pathog. Dis..

[30] Mughini-Gras L., Smid J.H., Wagenaar J.A., de Boer A.G., Havelaar A.H., Friesema I.H., French N.P., Busani L., van Pelt W. (2012). Risk factors for campylobacteriosis of chicken, ruminant, and environmental origin: A combined case-control and source attribution analysis. PLoS ONE.

[31] Dearlove B.L., Cody A.J., Pascoe B., Méric G., Wilson D.J., Sheppard S.K. (2016). Rapid host switching in generalist *Campylobacter* strains erodes the signal for tracing human infections. ISME J..

[32] Ocejo M., Oporto B., Hurtado A. (2019). Occurrence of *Campylobacter jejuni* and *Campylobacter coli* in Cattle and Sheep in Northern Spain and Changes in Antimicrobial Resistance in Two Studies 10-years Apart. Pathogens.

[33] Ocejo M., Oporto B., Lavín J.L., Hurtado A. (2021). Whole genome-based characterisation of antimicrobial resistance and genetic diversity in *Campylobacter jejuni* and *Campylobacter coli* from ruminants. Sci. Rep..

[34] Bianchini V., Borella L., Benedetti V., Parisi A., Miccolupo A., Santoro E., Recordati C., Luini M. (2014). Prevalence in bulk tank milk and epidemiology of *Campylobacter jejuni* in dairy herds in Northern Italy. Appl. Environ. Microbiol..

[35] Wu Z., Sippy R., Sahin O., Plummer P., Vidal A., Newell D., Zhang Q. (2014). Genetic diversity and antimicrobial susceptibility of Campylobacter jejuni isolates associated with sheep abortion in the United States and Great Britain. J. Clin. Microbiol..

[36] Yaeger M.J., Sahin O., Plummer P.J., Wu Z., Stasko J.A., Zhang Q. (2021). The pathology of natural and experimentally induced *Campylobacter jejuni* abortion in sheep. J. Vet. Diagn. Investig..

[37] Gülmez Sağlam A., Akça D., Çelebi Ö., Büyük F., Çelik E., Coşkun M.R., Şahin M., Otlu S. (2019). Isolation and molecular identification of *Campylobacter* spp. from vaginal swab sample obtained from sheep herds with abort history. Kafkas Univ. Vet. Fak. Derg..

[38] Guyard-Nicodème M., Rivoal K., Houard E., Rose V., Quesne S., Mourand G., Rouxel S., Kempf I., Guillier L., Gauchard F. (2015). Prevalence and characterization of *Campylobacter jejuni* from chicken meat sold in French retail outlets. Int. J. Food Microbiol..

[39] Thépault A., Guyard-Nicodème M., Rose V., Quesne S., Queguiner M., Houard E., Mégraud F., Rivoal K., Chemaly M. (2018). A representative overview of the genetic diversity and lipooligosaccharide sialylation in *Campylobacter jejuni* along the broiler production chain in France and its comparison with human isolates. Int. J. Food Microbiol..

[40] Sheppard S.K., Dallas J.F., MacRae M., McCarthy N.D., Sproston E.L., Gormley F.J., Strachan N.J., Ogden I.D., Maiden M.C., Forbes K.J. (2009). *Campylobacter* genotypes from food animals, environmental sources and clinical disease in Scotland 2005/6. Int. J. Food Microbiol..

[41] Denis M., Nagard B., Rose V., Bourgouin K., Cutimbo M., Kerouanton A. (2017). No clear differences between organic or conventional pig farms in the genetic diversity or virulence of *Campylobacter coli* isolates. Front. Microbiol..

[42] Thakur S., Morrow W.E., Funk J.A., Bahnson P.B., Gebreyes W.A. (2006). Molecular epidemiologic investigation of Campylobacter coli in swine production systems, using multilocus sequence typing. Appl. Environ. Microbiol..

[43] Miller W.G., Englen M.D., Kathariou S., Wesley I.V., Wang G., Pittenger-Alley L., Siletz R.M., Muraoka W., Fedorka-Cray P.J., Mandrell R.E. (2006). Identification of host-associated alleles by multilocus sequence typing of *Campylobacter coli* strains from food animals. Microbiology.

[44] Litrup E., Torpdahl M., Nielsen E.M. (2007). Multilocus sequence typing performed on *Campylobacter coli* isolates from humans, broilers, pigs and cattle originating in Denmark. J. Appl. Microbiol..

[45] Egger R., Korczak B.M., Niederer L., Overesch G., Kuhnert P. (2012). Genotypes and antibiotic resistance of *Campylobacter coli* in fattening pigs. Vet. Microbiol..

[46] Di Donato G., Marotta F., Nuvoloni R., Zilli K., Neri D., Di Sabatino D., Calistri P., Di Giannatale E. (2020). Prevalence, Population Diversity and Antimicrobial Resistance of *Campylobacter coli* Isolated in Italian Swine at Slaughterhouse. Microorganisms.

[47] French N., Barrigas M., Brown P., Ribiero P., Williams N., Leatherbarrow H., Birtles R., Bolton E., Fearnhead P., Fox A. (2005). Spatial epidemiology and natural population structure of *Campylobacter jejuni* colonizing a farmland ecosystem. Environ. Microbiol..

[48] Sopwith W., Birtles A., Matthews M., Fox A., Gee S., Painter M., Regan M., Syed Q., Bolton E. (2008). Identification of potential environmentally adapted *Campylobacter jejuni* strain, United Kingdom. Emerg. Infect. Dis..

[49] Hock L., Herold M., Walczak C., Schoos A., Penny C., Cauchie H.M., Ragimbeau C. (2023). Environmental dynamics of *Campylobacter jejuni* genotypes circulating in Luxembourg: What is the role of wild birds?. Microb. Genom..

